# Alterations in the gut microbiome and metabolism with doxorubicin-induced heart failure severity

**DOI:** 10.3389/fmicb.2024.1348403

**Published:** 2024-12-24

**Authors:** Qian Wang, Meihua Liu, Tianpei Liu, Long Li, Chenyang Wang, Xiaolin Wang, Shuling Rong, Xuedong Zhou

**Affiliations:** ^1^Department of Cardiology, The Second Hospital of Shanxi Medical University, Taiyuan, China; ^2^Academy of Medical Sciences, The Shanxi Medical University, Taiyuan, China; ^3^Department of Neonatology, The Second Hospital of Shanxi Medical University, Taiyuan, China; ^4^State Key Laboratory of Oral Diseases, National Clinical Research Center for Oral Diseases, Department of Cariology and Endodontics, West China Hospital of Stomatology, Sichuan University, Chengdu, China

**Keywords:** doxorubicin, heart failure, gut microbiota, 16S rRNA gene sequencing, metabolomics, multivariate analysis

## Abstract

**Objective:**

This study aimed to explore the changes in gut microbiota and its metabolites in different pathophysiological stages of doxorubicin (DOX)-induced heart failure (DIHF) and the relationship between gut microbiota and metabolites in various degrees of DIHF.

**Materials and methods:**

C57BL/6 J mice were injected intraperitoneally with 5 mg/kg of DOX once a week for 5 consecutive weeks. At different times after injection, the cardiac function and histopathological analysis was conducted, the serum levels of creatine kinase (CK), CK-MB, lactic dehydrogenase, and cardiac troponin T were determined. 16S rRNA gene sequencing of feces and the nontargeted metabolomics analysis of serum were performed. Multi-omics analyses were used to explore the correlation between gut microbiota and serum metabolites.

**Results:**

The results showed that DOX caused cardiac contractile dysfunction and left ventricular (LV) dilation. The levels of myocardial enzymes significantly increase in 3 and 5 weeks after DOX injection. DOX-treated mice showed significant differences in the composition and abundance of gut microorganisms, and the levels of serum metabolites at different times of treatment. Multi-omics analyses showed that intestinal bacteria were significantly correlated with the differential metabolites. Some bacteria and metabolites can be used as biomarkers of DIHF (AUC > 0.8). KEGG analyses showed the involvement of different metabolic pathways in various degrees of DIHF.

**Conclusion:**

Marked differences were found in the composition and abundance of gut microorganisms, the levels of serum metabolites and metabolic pathways in different degrees of DIHF. The intestinal bacteria were significantly correlated with differential metabolites in different degrees of DIHF. The gut microbiota may serve as new targets for the treatment of DIHF.

## 1 Introduction

Doxorubicin (DOX) is a representative anthracycline antibiotic with a broad antitumor spectrum and potent action, and is widely used for treating various solid tumors and hematologic malignancies (Arcamone et al., 1969). However, DOX suffers from severe dose-dependent cardiotoxicity that leads to irreversible congestive heart failure (HF), thus limiting its clinical application (Yu et al., 2018; Wang et al., 2021). Research has shown that the DOX can induce myocardial toxicity via various mechanisms such as oxidative stress, iron metabolism, inflammation, and Ca^2+^overload (Yu et al., 2020; Christidi and Brunham, 2021; Zhu et al., 2010; Arai et al., 2000). Thus, a variety of strategies including limitation of cumulative DOX doses, use of antioxidant drugs, and standard anti-heart failure therapies have been proposed to ameliorate DOX-induced HF (DIHF), however, these strategies is not much satisfying (Huang et al., 2022). Therefore, it is crucial to better understand the pathological mechanisms and established novel strategies to prevent and treat DOX-induced myocardial toxicity.

Gut microbes has been found to play an essential role in the pathophysiological process of atherosclerosis, dyslipidemia, hypertension, heart failure, coronary heart disease (CAD), and obesity through immune response, inflammatory response, and oxidative stress (Kazemian et al., 2020; Rahman et al., 2022). The potential role of gut microbiota in DIHF has received widespread attention in recent years (Huang et al., 2022; Fan et al., 2023). The composition imbalance and functional changes of the gut microbiota can be one of the underlying etiological mechanisms of DIHF (Huang et al., 2022). However, how the gut microbiota change in different pathophysiological stages of HF under the action of DOX is still not fully clarified.

The gut microbiota related metabolites have been implicated in the progression of cardiovascular diseases (CVDs). Gut microbes provide nutrients and energy to the host through the digestion of the ingested food and metabolism, which subsequently producing biologically active signaling molecules to maintain the body’s health. Diseases can also be triggered when the metabolism of the intestinal flora is disturbed. Several studies have reported on the relationship between metabolites of some intestinal microorganisms and cardiovascular diseases (Kazemian et al., 2020; Rahman et al., 2022; Liu et al., 2019; Yousuf et al., 2022) However, whether gut microorganisms regulate the metabolites of DIHF is still not fully clarified.

We constructed a model of HF established by intraperitoneal injection of DOX. Comprehensive analyses of their gut microbiota and metabolomic profiles were conducted using 16S amplicon sequencing and liquid chromatography combined with tandem mass spectrometry (LC–MS/MS) to address the aforementioned issues. Furthermore, we assessed the correlation between gut microorganisms and serum metabolites and explored whether gut microbiota regulated the metabolism in DOX-treated mice. In addition, we screened the biomarkers of microflora and metabolites related to DIHF. This study might provide new targets and new ideas for treating DIHF.

## 2 Materials and methods

### 2.1 Materials

DOX was purchased from Shenzhen Wanle Pharmaceutical Co., Ltd. (Wanle, Shenzhen, China). The kits for lactate dehydrogenase (LDH), creatine kinase (CK), creatine kinase isozyme (CK-MB), and cardiac troponin T (cTnT) were acquired from Jiancheng Technology Co. (Nanjing, China).

### 2.2 Animals and treatment

The animal experiments in this study was approved by the Institutional Animal Care and Use Committee at Second Hospital of Shanxi Medical University (DW2022070) and conformed to the Guide for the Care and Use of Laboratory Animals published by the US National Institute of Health. Healthy male C57BL/6 J mice (aged 8 weeks) were purchased from the Shanxi Medical University (Taiyuan, China, SCXK Jin 2019-0004) and housed in the Specified Pathogen Free (SPF) Laboratory of the Second Hospital of Shanxi Medical University (Taiyuan, China, SYXK Jin 2021-0001). All C57BL/6 J mice were randomly divided into control group (*n* = 13), DOX-1w group (DOX treatment for 1 week, *n* = 13), DOX-3w group (DOX treatment for 3 week, *n* = 13), and DOX-5w group (DOX treatment for 5 week, *n* = 13) after 1 week adaptive feeding. DOX was configured with a concentration of 1 mg/mL, and the mice in the DOX-1w, DOX-3w, and DOX-5w groups were injected intraperitoneally with DOX (5 mg/kg) once a week for 1 week, 3 and 5 consecutive weeks, respectively. The cumulative doses of DOX in DOX-1w, DOX-3w and DOX-5w groups were 5 mg/kg, 15 mg/kg, and 25 mg/kg, respectively. The mice in the control group were intraperitoneally injected with an equal volume of normal saline once a week for 5 weeks. After the treatment of DOX, ten mice survived in the DOX-5w group, 12 mice survived in the DOX-3w group, and 13 mice survived in control and DOX-1w groups.

### 2.3 Cardiac echocardiography

At 1, 3, and 5 week after the injection of DOX, the mice were anesthetized with 1.5% isoflurane, and echocardiograms were performed with a MX-550D probe at the papillary muscle level near the sternum of mice for five consecutive cardiac cycles with the Vevo 3100 high-resolution imaging system (Fujifilm VisualSonics, Canada). The left ventricular ejection fraction (LVEF), left ventricular short-axis shortening rate (LVFS), left ventricular internal diameter at end-systole (LVIDs), and left ventricle posterior wall thickness at end-systole (LVPWs) were recorded.

### 2.4 Determination of serum biochemical indices

The serum levels of LDH, CK, CK-MB, and cTnT are direct signals of myocardium damage. The serum of mice was obtained by centrifugation at 3,000 rpm for 10 min. The serum levels of LDH, CK, CK-MB, and cTnT were detected using relevant commercial kits following the manufacturer’s instructions (Nanjing Jiancheng Bioengineering Institute, China). The nontargeted metabonomics was detected using LC-MS/MS.

### 2.5 Histological examination

After performing cardiac echocardiography and extracting blood, the mice were sacrificed with intravenous pentobarbital sodium (100 mg/kg) at the end of study.

The LV myocardial tissue was harvested, fixed in 4% formaldehyde, subsequently embedded in paraffin, and then routinely sectioned. Then hematoxylin–eosin (H-E) staining was performed, and Masson trichome staining was used to determine myocardial fibrosis.

### 2.6 Collection of fecal samples and DNA extraction

The feces were removed from mice in a sterile state and immediately placed in a sterile tube. Approximately 200–500 mg was taken from each tube, frozen in liquid nitrogen for 15 min, and then immediately stored in a refrigerator at −80°C. DNA was extracted from the gut microbes using a PowerFecal DNA isolation kit (MAGEN, Guangzhou, China) following the manufacturer’s protocols. DNA concentration and purity were assessed using a NanoDrop 2000 micro-ultraviolet spectrophotometer (Thermo Fisher Scientific, MA, United States) and quantified with a Qubit fluorometer using a Qubit dsDNA BR assay kit (Invitrogen, CA, United States). DNA quality was checked by running an aliquot on 1% agarose gel. The detection and analysis were performed by Shenzhen Huada Gene Technology Co., Ltd. (Shenzhen, China).

### 2.7 Gut microbiota 16S rRNA sequencing and data analysis

Variable regions, V3–V4, of the bacterial 16S rDNA gene were amplified with polymerase chain reaction primers, 338F (5′-ACTCCTACGGGAGGCAGCAG-3′) and 806R (5′-GGACT ACHVGGGTWTCTAAT-3′), and the products were purified using Agencourt AMPure XP beads and eluted with the elution buffer. The libraries were qualified using an Agilent Technologies 2100 bioanalyzer (Agilent Technologies, CA, United States) and sequenced using an Illumina HiSeq 2500 platform (BGI, Shenzhen, China), following the manufacturer’s protocols. Then, paired-end reads were generated. The raw reads were filtered and paired-end reads were tagged for clustering into operational taxonomic units (OTUs) with a cutoff value of 97%.

The OTUs representative sequences were taxonomically classified using Ribosomal Database Project Classifier v.2.2 with a minimum confidence threshold of 0.6 and trained on the Greengenes database v201305 using Quantitative Insights Into Microbial Ecology (QIIME) v1.8.0. (United States). Alpha and beta diversities were estimated using Mothur (v1.31.2) (https://www.mothur.org, United States) and QIIME (v1.8.0), respectively. R package 3.4.1 and R package “gg plots” were used to plot various clusters. The Wilcoxon test was used to ascertain significant species with R v3.4.1.

### 2.8 Serum nontargeted metabolomics analysis

The untargeted metabolomics analysis was conducted using LC–MS/MS. A Q-Exactive high-resolution mass spectrometer (Thermo Fisher Scientific, MA, United States) was used to collect data from both positive ions (pos) and negative ions (neg) to improve metabolite coverage. LC–MS/MS data were processed using the Compound Discoverer 3.1 software (Thermo Fisher Scientific, MA, United States), mainly including peak extraction, peak alignment, and compound identification. Data pre-processing, statistical analysis, metabolite classification annotations, and functional annotations were executed using the metabolomics R package metaX (Shenzhen Huada Gene Technology, China) and the metabolome bioinformatic analysis pipeline. The multivariate raw data were dimensionally reduced using Principal Component Analysis (PCA) to analyze the groupings, trends (intra-and inter-group similarities and differences), and outliers of the observed variables in the dataset (whether an abnormal sample was present). We used partial least squares–discriminant analysis (PLS-DA) to combine the variable importance in projection (VIP) values of the first two principal components of the model with the variability analysis, fold change, and Student *t* test to screen for differential metabolites. The metabolic pathway enrichment analysis of differential metabolites was performed based on the Kyoto Encyclopedia of Genes and Genomes (KEGG) database. A *p* value <0.05 was considered statistically significant.

### 2.9 Statistical analysis

The experimental data were analyzed using SPSS 26.0 and GraphPad Prism 8.0 statistical software. Prior to analysis, normality and homogeneity of variance tests were conducted on the data. When the data met the criteria of normal distribution and variance homogeneity, data were expressed as mean ± standard deviation (SD), and the *t*-test was employed. In cases where the data did not conform to normal distribution and variance homogeneity, the Wilcoxon rank-sum test was used. A *p* value <0.05 was considered statistically significant.

## 3 Results

### 3.1 Echocardiography and serum cardiac enzymes

**Figure 1:**
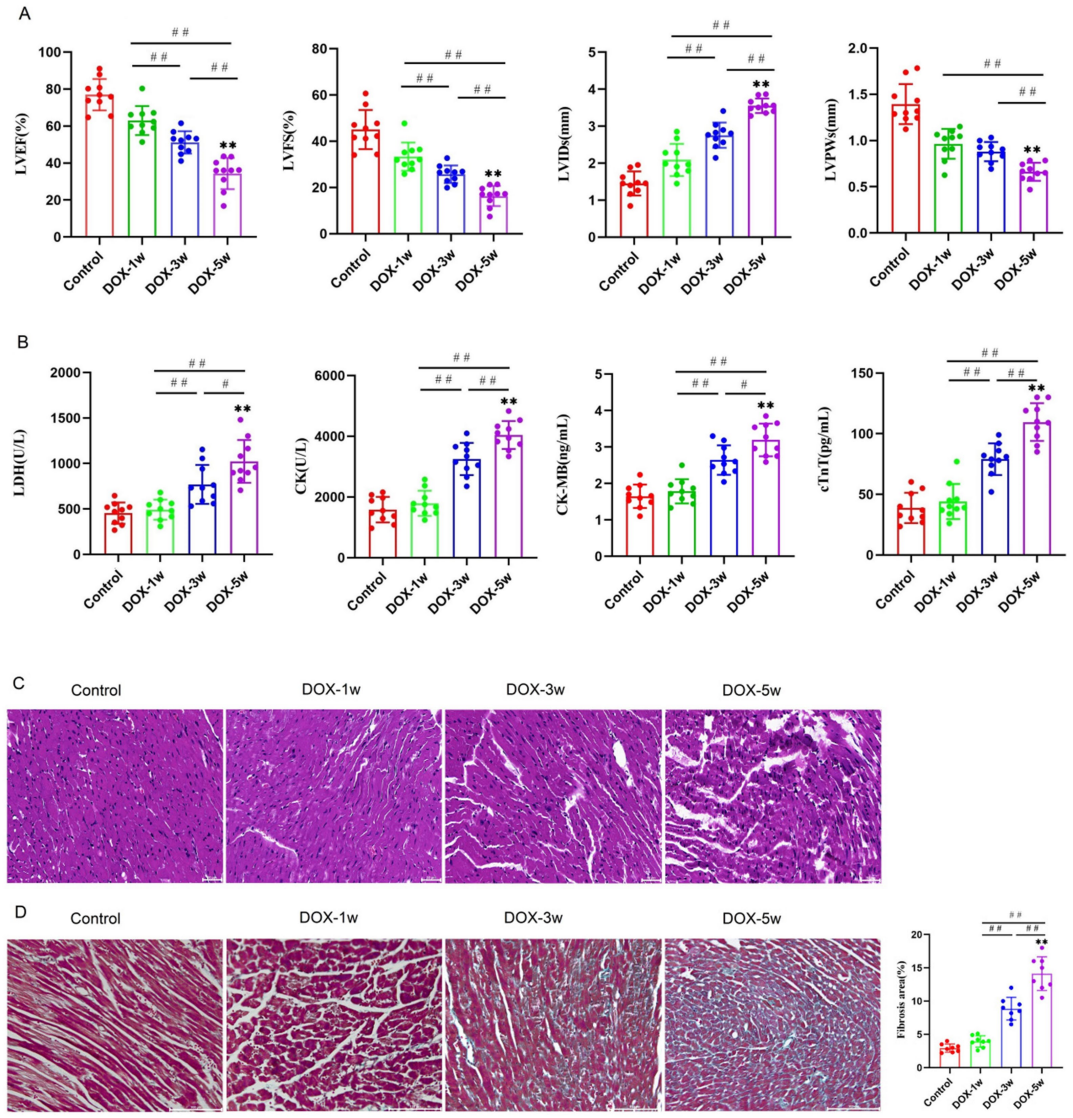
DOX-induced cardiac dysfunction, myocardial necrosis (increase in the levels of myocardial enzymes) and histological injury. **(A)** Comparison of echocardiography parameters. Effects of DOX on the echocardiography parameters in different groups. *n* = 10. **(B)** Serum levels of CK, CK-MB, LDH, and cTnT in different groups. *n* = 10. **(C)** Representative photographs of H&E staining (scale bar = 50 μm). **(D)** Representative photographs and quantitative data of Masson trichrome staining of heart sections (scale bar = 100 μm). *n* = 8. EF%, Left ventricular ejection fraction; FS%, Left ventricular fractional shortening; LVIDs, Left ventricular internal diameter at end-systole; LVPWs, Left ventricle posterior wall thickness at end-systole. DOX, Doxorubicin; CK, Creatine kinase; CK-MB, Creatine kinase-MB; LDH, Lactic dehydrogenase; and cTnT, Cardiac troponin T. Data are presented as mean ± SD. ^**^p < 0.01 vs. Control group; ^#^*p* < 0.05, ^##^*p* < 0.01 vs. DOX-1w or DOX-3w.

Echocardiography results illustrated that the LVEF, LVFS and LVPWs significantly decreased, and LVIDs significantly increased in the DOX-5w group compared with the control, DOX-1w, and DOX-3w groups. Moreover, LVEF, LVFS and LVPWs in the DOX-3w group were significantly lower than those in the DOX-1w group, whereas LVIDs in the DOX-3w group were significantly higher than those in the DOX-1w group (*p* < 0.05) (Figure 1A). These results suggested that the cardiac function deteriorate with the increase in the accumulation of drugs in mice.

As depicted in Figure 1B, LDH, CK, CK-MB, and cTnT were significantly increased in the DOX-5w group compared with the control group, DOX-1w group and the DOX-3w group (*p* < 0.05). Moreover, LDH, CK, CK-MB, and cTnT were significantly increased in the DOX-3w group compared with the DOX-1w group (*p* < 0.05).

### 3.2 Histological examination of mice heart

HE staining was performed to further clarify the histological changes in the heart caused by DOX. As shown in Figure 1C, we observed that the myocardial fiber of the control group mice were distributed regularly and tightly. The myocardial fiber arrangement was partially disordered in DOX-1w mice. The myocardium was slightly congested, the disorder of myocardial fiber arrangement was serious, edema was visible, and the plasma and fibrin in the vein were exuded in DOX-3w mice. Myocardial congestion was serious, myoplasm was dissolved, myocardial texture was not clear, and obvious swelling, degeneration, or necrosis of myocardial cells could be seen in the DOX-5w mice. Myocardial fibrosis was detected by Masson’s trichrome staining. There was no apparent myocardial fibrosis in the DOX-1w group, but the myocardial fibrosis significantly increased in DOX-3w group compared with the DOX-1w group. The most severe myocardial fibrosis was observed in the DOX-5w group (Figure 1D). The aforementioned results suggested that the cardiac toxicity caused by DOX increased with the increase in the accumulation of drugs in mice, and the DOX-5w group had the most serious myocardial damage.

### 3.3 Amplicon sequencing results of mice gut microbiota

#### 3.3.1 Analysis of bacterial diversity

Alpha diversity is used to describe the diversity of microbial communities within a sample. No significant differences were observed in alpha diversity between different groups. This suggested no difference in microbial community diversity within samples. Beta diversity is used to describe the similarity or dissimilarity in species composition of gut microbial communities in different study subject groups.

**Figure 2:**
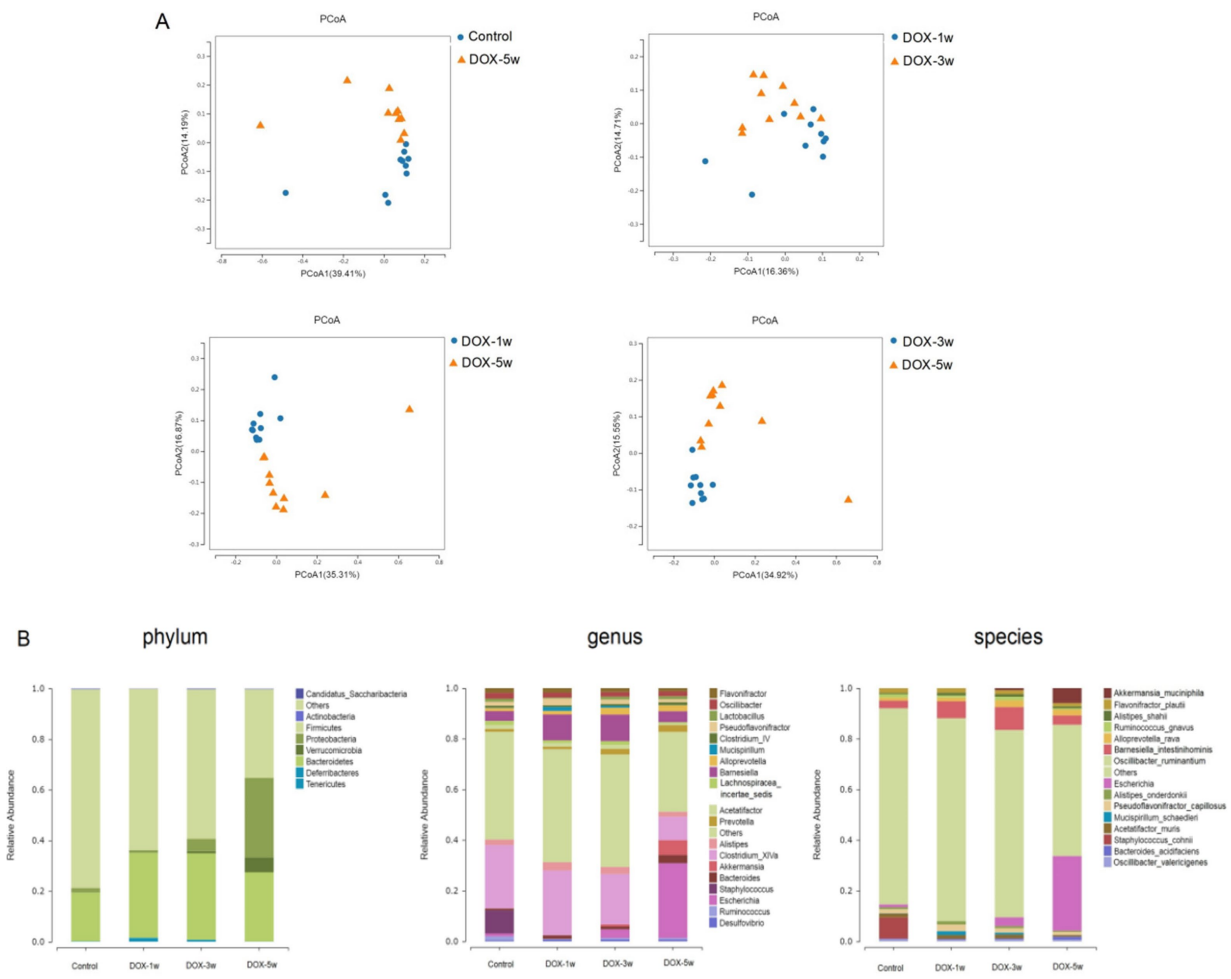
Beta diversity analysis of intestinal flora and species composition distribution. **(A)** PCoA plot based on the unweighted UniFrac distance of gut microbiota samples from Control vs. DOX-5 group (*p* = 0.0035), DOX-3 vs. DOX-1 group (*p <* 0.0001), DOX-5 vs. DOX-1 group (*p <* 0.0001), DOX-5 vs. DOX-3 group (*p <* 0.0001). **(B)** Species composition distribution at the phylum, genus, and species levels in different groups. *n* = 10.

Principal Coordinate Analysis (PCoA) of weighted and unweighted UniFrac distances is used to assess the beta diversity. The unweighted-unifrac-based beta diversity significantly differed between the control and DOX-5w groups (*p* = 0.0035), the DOX-1w and DOX-3w groups (*p* < 0.0001), DOX-1 and DOX-5 groups (*p* < 0.0001), and the DOX-3w and DOX-5w groups (*p* < 0.0001) (Figure 2A).

These results suggested that with the extension of DOX treatment time and the cumulative measurement increased, the difference in the composition of intestinal microorganisms between different DOX treatment groups increased significantly.

#### 3.3.2 Microbial composition analysis

Based on the species annotation analysis of OTUs, we found eight phylum of fecal flora sequence in the control, DOX-1w, DOX-3w, and DOX-5w groups, we found that the dominant phyla of the four groups were identical, which included *Firmicutes*, *Bacteroidota*, *and Proteobacteria*. At the phylum level, the relative abundances of *Firmicutes*, *Bacteroidetes*, and *Proteobacteria* were > 93% in all groups. *Firmicutes* was most higher in the DOX-1w group (approximately 63.62%), but decreased to 58.99 and 34.92% in the DOX-3w and DOX-5w groups, respectively. In contrast, *Proteobacteria* increased from 1.75% in the DOX-1w group to 4.81% in the DOX-3w, and even up to 31.41% in the DOX-5w group (Figure 2B).

The species composition bar chart at the genus level shows the top 20 species with the highest abundance (Figure 2B). The genus *Prevotella* increased from 0.98% in the DOX-1w group to 2.20 and 2.71% in the DOX-3w and DOX-5w groups, respectively, and the genus *Bacteroides* increased from 1.4 and 1.1% in DOX-1w and DOX-3w groups to 3.1% in the DOX-5w group.

The species composition bar chart at the species level shows the top 20 species with the highest abundance (Figure 2B). The species *Bacteroides acidifaciens* from 0.38% in the DOX-3w group to 1.10% in DOX-5w group, the species *Escherichia* from 0.008% in the DOX-1w group to 3.4 and 29% in DOX-3w and DOX-5w groups; While species *Akkermansia muciniphila* from 0.02% in the DOX-1w group to 0.08% in DOX-3w group, and even up to 5.8% in the DOX-5w group.

#### 3.3.3 Differential analysis of intestinal microflora

Based on the abundance profiles, the features with significant differential abundance across groups were determined using the Wilcoxon rank-sum test. At the phylum level, the abundance of *Verrucomicrobia* in the DOX-3w group significantly increased compared with the DOX-1w group (*p* < 0.05). Also, the abundance of *Proteobacteria* significantly increased in the DOX-5w group compared with the control group and DOX-1w group (*p* < 0.05), while the abundance of *Firmicutes* in the DOX-5w group significantly decreased compared with the control group and DOX-1w group (*p* < 0.05). No difference was found in the DOX-5w group compared with the DOX-3w group. These results showed that the intestinal flora of mice changed significantly at the phylum level with the increase in the DOX dose (Supplementary Table S1).

At the genus level, 12 intestinal microorganisms were screened in the DOX-5w group compared with the control group, among which, the abundance of *Bifidobacterium, Romboutsia, Escherichia, Mycoplasma, Turiciactor, Clostridium XVIII, Butyrisimonas*, and *Clostridium sensu stricto* significantly increased, and that of *Roseburia, Asaccharacter, Gemmiger*, and *Peptococcus* significantly decreased.

**Figure 3:**
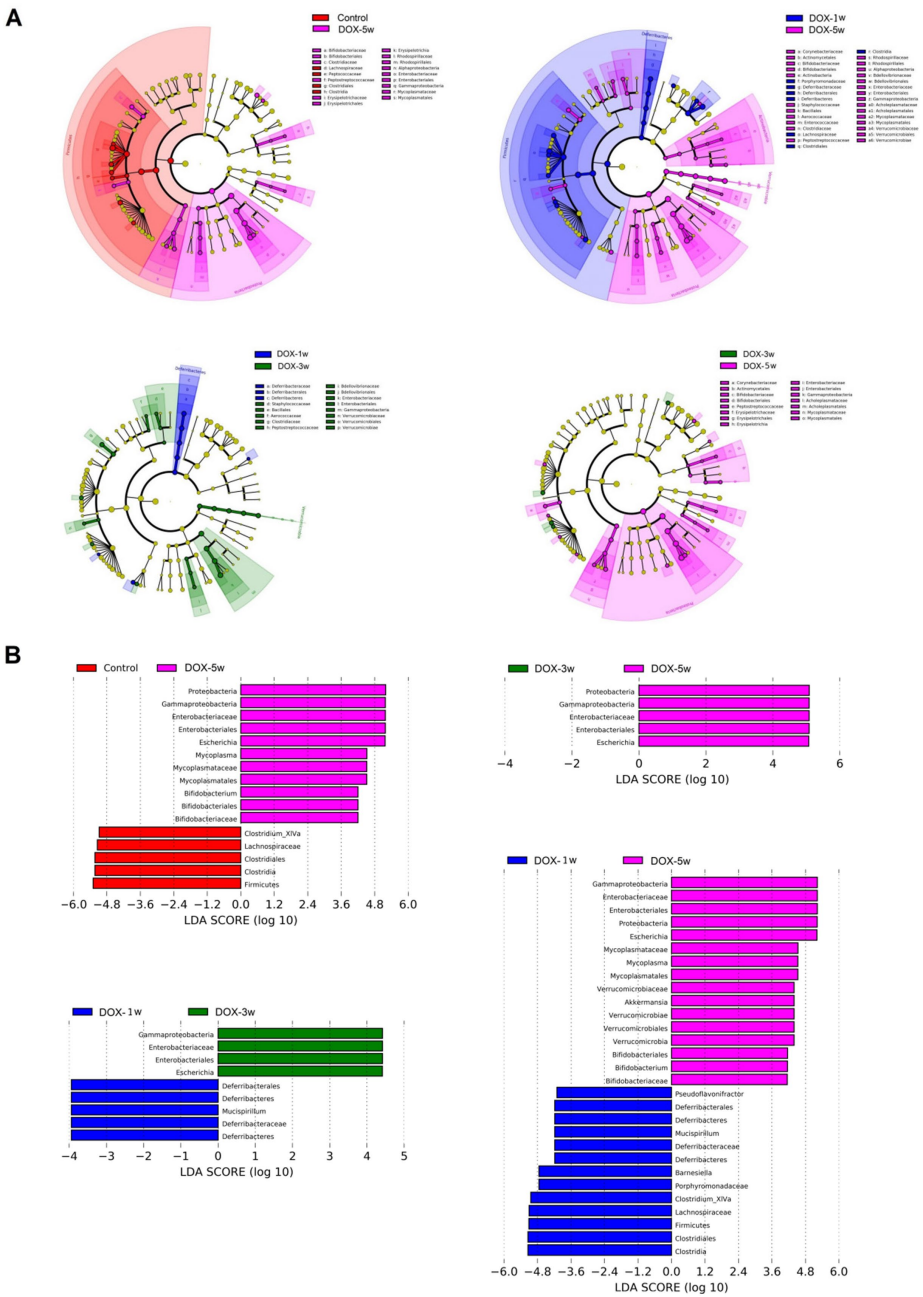
LEfSe analysis between different groups. **(A)** LEfSe cladogram from phylum to genus levels between different groups. Different color nodes represent the microbiota that are significantly enriched in the corresponding group and have significant differences between the different groups. **(B)** Histogram analyzed by LEfSe showed that the microbiota with significant differences between the different groups. (LDA score > 4.0). *p* < 0.05. *n* = 10. LDA, Linear discriminant analysis; LEfSe, Linear discriminant analysis effect size.

Moreover, at the genus level, compared with the DOX-1w group, one intestinal microorganisms were screened in the DOX-3w group, and the abundance of *Clostridium sensu stricto* significantly increased in the DOX-3w group. However, 14 intestinal microorganisms were screened in the DOX-5w group compared with the DOX-1w group, among which, the abundance of *Turicibacter*, *Escherichia*, *Butyricimonas*, *Klebsiella*, *Romboutsia*, *Mycoplasma*, *Staphylococcus*, *Bifidobacterium*, and *Clostridium sensu stricto* significantly increased, while *Roseburia, Mucispirillum, Butyricicoccus, Clostridium XlVa*, and *Rikenella* significantly decreased. Compared with the DOX-3w group, DOX-5w group screened 6 intestinal microorganisms, among which, the abundance of *Turiciactor, Bifidobacterium, Butyricomimonas, Faecalibacterium, Mycoplasma*, and *Klebsiella* significantly increased. These results indicated that the gut microbiota significantly changed at the genus level with the increase in the DOX dose (Supplementary Table S2). Linear discriminant analysis effect size (LEfSe) was used to detect biomarkers (Figure 3).

At the species level, nine different bacteria were screened in the DOX-5w group compared with the control group, among which, the abundance of *Bifidobacterium pseudolongum, Romboutsia sedimentorum, Escherichia, Mycoplasma hyorhinis, Turicibacter sanguinis*, and *Clostridium. cocleatum* significantly increased and that of *Roseburia faecis, Faecalicoccus pleomorphus, and Asaccharobacter celatus* significantly reduced.

At the species level, no difference was observed in the DOX-3w group compared with the DOX-1w group. However, 13 intestinal microorganisms were screened in the DOX-5w group compared with the DOX-1w group, among which, the abundance of *Clostridium disporicum, Turicibacter sanguinis, Escherichia, Butyricimonas virosa, Romboutsia sedimentorum, Bacteroides caccae, Staphylococcus cohnii, Mycoplasma hyorhinis, Bifidobacterium pseudolongum, and Bacteroides nordii significantly increased*, while *Roseburia faecis, Mucispirillum schaedleri*, and *Butyricicoccus pullicaecorum* significantly decreased. Compared with the DOX-3w group, the abundance of *Turicibacter sanguinis, Bacteroides caccae, Bifidobacterium pseudolongum, Faecalibacterium prausnitzii*, and *Mycoplasma hyorhinis* significantly increased and that of *Roseburia faecis* significantly decreased in the DOX-5w group (Supplementary Table S3).

### 3.4 Analysis of untargeted metabolomics results in mice serum

#### 3.4.1 PLS-DA analysis

**Figure 4:**
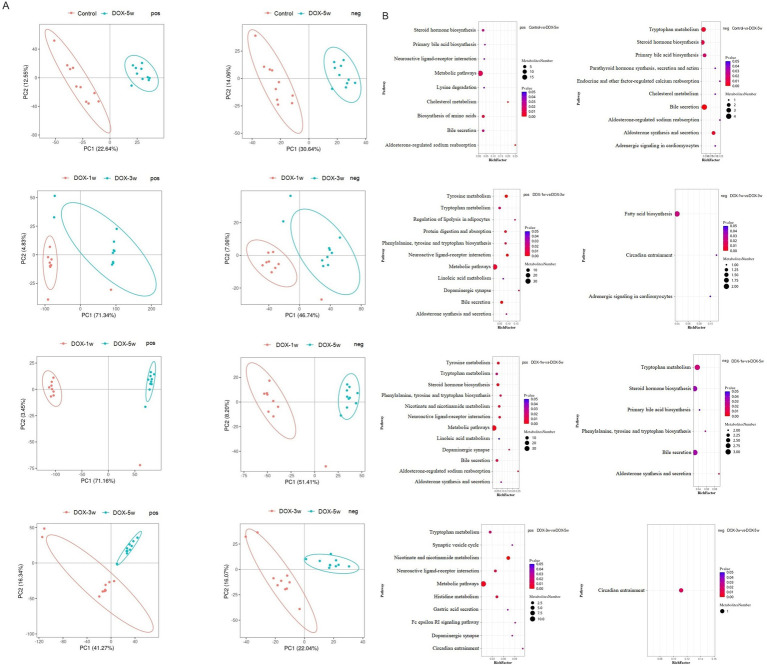
PLS-DA score graph of positive and negative ion modes and metabolic pathway enrichment analysis. **(A)** PLS-DA score graph of positive and negative ion modes in different groups. **(B)** Bubble plots for metabolic pathway enrichment analysis. *n* = 10, *p* < 0.05. pos, positive ion mode; neg, negative ion mode.

Partial least squares–discriminant analysis (PLS-DA) uses partial least squares regression to establish the relationship model between metabolite expression and sample category. The analysis results are demonstrated in Figure 4A. Significantly different separation trends of serum metabolites were observed among three groups (DOX-1w group, DOX-3w group, and DOX-5w group), indicating significant differences in metabolites between different treatment groups.

#### 3.4.2 Serum differential metabolites

We conducted nontargeted LC–MS/MS metabolomics analysis on the serum samples from different groups. We observed significantly different metabolite profiles based on the PLS-DA models of metabolite profiling data. A total of 149 substances were upregulated and 219 substances were downregulated in the DOX-5w group compared with the control group, among which, the levels of tauroursodeoxycholic acid (TUDCA), deoxycholic acid (DCA), indole-3-acrylic acid, cystine, and l-histidine significantly increased and that of gluconic acid, aceglutamide, and creatine significantly decreased. Further, 269 substances were upregulated and 348 substances were downregulated in the DOX-3w group compared with the DOX-1w group, among which, the levels of TUDCA, docosahexaenoic acid, l-tryptophan, and d-ornithine significantly increased and that of his-tyr, l-gulonolactone and 3-hydroxysebacic acid significantly decreased. Also, 365 substances were upregulated and 409 substances were downregulated in the DOX-5w group compared with the DOX-1w group, among which, the levels of indole-3-acrylic acid, jasmonic acid, arg-gln, and d-ornithine significantly increased and that of dl-arginine, leu-leu, and sphingosine-1-phosphate (d16:1) significantly decreased. Moreover, 112 substances were upregulated and 51 substances were downregulated in the DOX-5w group compared with the DOX-3w group, among which, the levels of cholic acid, nicotinic acid, and indole-3-acrylic acid significantly increased and that of lysolecithin, prohydrojasmon, and 3,10-dihydroxydecanoic acid significantly decreased (VIP ≥ 1, level ≤ 4, fold-change ≥1.2 or ≤ 0.83, and *p* value <0.05 were used as the screening conditions for differential metabolites).

We performed receiver operating characteristic (ROC) analysis on the metabolites identified in different groups to find the metabolic biomarkers of varying degrees of HF in serum. The metabolites with predictive value in the control vs. DOX-5w group were as follows: tyrosyltyrosine (AUC = 0.94), aldosterone (AUC = 0.90), DCA (AUC = 0.87), cystine (AUC = 0.86), n-acetyl-L-phenylalanine (AUC = 0.96), trimethylamine N-oxide (TMAO, AUC = 0.93), taurocholic acid (AUC = 0.90), indole-3-acrylic acid (AUC = 0.91), l-histidine (AUC = 0.89), and TUDCA (AUC = 0.81), among others (Supplementary Table S4).

The metabolites with predictive value in the DOX-1w vs. DOX-3w group were as follows: daminozide (AUC = 0.99), lycopsamine (AUC = 0.99), phthalic acid (AUC = 0.97), choline (AUC = 0.94), taurocholic acid (AUC = 0.88), l-tryptophan (AUC = 0.84), and TUDCA (AUC = 0.92), among others (Supplementary Table S5).

The metabolites with predictive value in the DOX-1w vs. DOX-5w group were as follows: d-ornithine (AUC = 0.99), 3-dehydroshikimate (AUC = 0.99), promegestone (AUC = 0.99), l-tryptophan (AUC = 0.91), and indole-3-acrylic acid (AUC = 0.99), among others (Supplementary Table S6).

The metabolites with predictive value in the DOX-3w vs. DOX-5w group were as follows: eslicarbazepine (AUC = 0.98), 4-hydroxybenzaldehyde (AUC = 0.90), indole-3-acrylic acid (AUC = 0.88), and cholic acid (AUC = 0.90) (Supplementary Table S7).

We could accurately distinguish the two groups and predict the disease by screening the metabolites with predictive value.

#### 3.4.3 Metabolic pathway analysis

Based on the KEGG database, metabolic pathway enrichment analysis was performed on different groups. Fourteen metabolic pathways with *p* value <0.05 were significantly enriched by differential metabolites in the DOX-5w group compared with the control group. These metabolic pathways mainly included aldosterone-regulated sodium reabsorption; biosynthesis of amino acid; bile secretion; primary bile acid biosynthesis; aldosterone synthesis and secretion; tryptophan metabolism; and adrenergic signaling in cardiomyocytes pathways, among others. The differential metabolites involved in these KEGG metabolic pathways mainly included taurocholic acid, l-histidine, cholic acid, glycocholic acid, DCA, aldosterone, corticosterone, corticosterone, and tetrahydrocortisone, among others (Figure 4B; Supplementary Table S8).

Fourteen metabolic pathways with *p* value <0.05 were significantly enriched by differential metabolites in the DOX-3w group compared with the DOX-1w group. These metabolic pathways mainly included bile secretion; phenylalanine, tyrosine and tryptophan biosynthesis; aldosterone synthesis and secretion; tryptophan metabolism; linoleic acid metabolism; and adrenergic signaling in cardiomyocytes pathways, among others. The differential metabolites involved in these KEGG metabolic pathways mainly included choline, dopamine, cortisol, taurocholic acid, cholate, 3-dehydroquinic acid, l-tryptophan, corticosterone, and arachidonic acid, among others (Figure 4B; Supplementary Table S9).

Thirteen metabolic pathways with *p*-value <0.05 were significantly enriched by differential metabolites in the DOX-5w group compared with the DOX-1w group. These metabolic pathways mainly included aldosterone-regulated sodium reabsorption; neuroactive ligand-receptor interaction; nicotinate and nicotinamide metabolism; bile secretion; phenylalanine, tyrosine and tryptophan biosynthesis; tryptophan metabolism; and aldosterone synthesis and secretion, among others. The differential metabolites involved in these KEGG metabolic pathways mainly included cortisone, cortisol, dopamine, tyramine, d-(−)-morphine, spermidine, glycocholate, d-(−)-quinic acid, l-tryptophan, 3-dehydroshikimate, corticosterone, desoxycortone, among others (Figure 4B; Supplementary Table S10).

Ten metabolic pathways with *p*-value <0.05 were significantly enriched by differential metabolites in the DOX-5w group compared with the DOX-3w group. These metabolic pathways mainly included nicotinate and nicotinamide metabolism; histidine metabolism; neuroactive ligand-receptor interaction; and gastric acid secretion, among others. The differential metabolites involved in these KEGG metabolic pathways mainly included histamine; urocanate; melatonin and nicotinic acid, among others (Figure 4B; Supplementary Table S11).

### 3.5 Multi-omics analysis revealed the relationship between gut microbiota and serum metabolites and cardiac enzymes in DOX-treated mice

We further examined the correlation between intestinal microbiota and serum metabolites to further determine whether intestinal microbes were involved in body metabolism. We performed Spearman correlation analysis on the identified intestinal microbiota and differential metabolites in serum between different groups. The results demonstrated that the differential microorganisms in the gut were significantly correlated with the identified differential metabolites with predictive value for DIHF.

**Figure 5:**
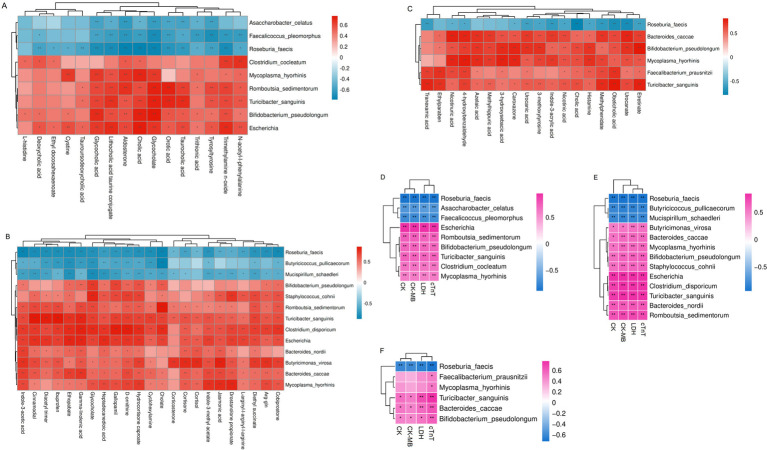
Multi-omics joint analysis. **(A)** Spearman’s correlation heatmap of gut microbiota and differential metabolites in the DOX-5w vs. Control group. **(B)** Spearman’s correlation heatmap of gut microbiota and differential metabolites in the DOX-5w vs. DOX-1w group. **(C)** Spearman’s correlation heatmap of gut microbiota and differential metabolites in the DOX-5w vs. DOX-3w group. **(D)** Spearman correlation heatmap of gut microbiota and serum levels of CK, CK-MB, LDH, and cTnT in the DOX-5w vs. Control group. **(E)** Spearman correlation heatmap of gut microbiota and serum levels of CK, CK-MB, LDH, and cTnT in the DOX-5w vs. DOX-1w group. **(F)** Spearman correlation heatmap of gut microbiota and serum levels of CK, CK-MB, LDH, and cTnT in the DOX-5w vs. DOX-3w group. The color blocks represent the correlation coefficient. The darker the color, the stronger the correlation between the microbial groups and the different metabolites. Red represents a positive correlation, and blue represents a negative correlation. CK, Creatine kinase; CK-MB, Creatine kinase-MB; LDH, Lactic dehydrogenase; cTnT, Cardiac troponin T. *n* = 10. ^*^p < 0.05, ^**^p < 0.01.

The differential metabolites aldosterone, cholic acid, cystine, DCA, ethyl docosahexaenoate, glycocholate, glycocholic acid, l-histidine, lithocholic acid taurine conjugate, n-acetyl-l-phenylalanine, orotic acid, taurocholic acid, TUDCA, TMAO, trithionic acid, and tyrosyltyrosine were positively correlated with bacterial strains *Bifidobacterium pseudolongum, Clostridium cocleatum, Escherichia, Mycoplasma hyorhinis, Romboutsia sedimentorum*, and *Turicibacter sanguinis*, and negatively correlated with *Asaccharobacter celatus, Roseburia faecis*, and *Faecalicoccus pleomorphus* in DOX-5w group compared with control group (Figure 5A).

The differential metabolites arg-gln, cholate, cortisol, cortisone, d-ornithine, daidzin, cyclohexylamine, corticosterone, diethyl succinate, drostanolone propionate, ethopabate, gamma-linolenic acid, glycocholate, heptadecanedioic acid, indole-3-acetic acid, indole-3-methyl acetate, jasmonic acid, indole-3-acrylic acid, and l-arginyl-l-arginyl-l-arginine were positively correlated with *Bacteroides caccae, Bacteroides nordii, Bifidobacterium pseudolongum*, *Butyricimonas virosa*, *Clostridium disporicum*, *Escherichia, Mycoplasma hyorhinis, Romboutsia sedimentorum, Staphylococcus cohnii*, and *Turicibacter sanguinis*, and negatively correlated with *Butyricicoccus pullicaecorum, Mucispirillum schaedleri*, and *Roseburia faecis* in DOX-5w group compared with DOX-1w group (Figure 5B).

The differential metabolites 3-hydroxysebacic acid, 3-methoxytyrosine, 4-hydroxy-3-methoxyphenylglycol sulfate, 4-hydroxybenzaldehyde, azelaic acid, caroxazone, cholic acid, ethylparaben, etretinate, histamine, indole-3-acrylic acid, methylhippuric acid, methylphenidate, nicotinic acid, nicotinuric acid, obeticholic acid, tranexamic acid, urocanate and urocanic acid were positively correlated with *Bacteroides caccae, Bifidobacterium pseudolongum, Faecalibacterium prausnitzii, Mycoplasma hyorhinis* and *Turicibacter sanguinis*, and negatively correlated with *Roseburia faecis* in DOX-5w group compared with DOX-3w group (Figure 5C).

The myocardial necrosis markers LDH, CK, CK-MB, and cTnT were positively correlated with *Bifidobacterium pseudolongum*, *Clostridium cocleatum*, Escherichia, *Mycoplasma hyorhinis*, Romboutsia sedimentorum, and *Turicibacter sanguinis*, and negatively correlated with *Asaccharobacter celatus*, *Roseburia faecis*, and Faecalicoccus pleomorphus in the DOX-5w group compared with the control group (Figure 5D).

The myocardial necrosis markers LDH, CK, CK-MB, and cTnT were positively correlated with *Bacteroides caccae*, *Bacteroides nordii*, *Bifidobacterium pseudolongum*, *Butyricimonas virosa*, *Clostridium disporicum*, *Escherichia*, *Mycoplasma hyorhinis*, *Romboutsia sedimentorum*, *Staphylococcus cohnii* and *Turicibacter sanguinis*, and negatively correlated with *Butyricicoccus pullicaecorum*, *Mucispirillum schaedleri* and *Roseburia faecis* in DOX-5w group compared with DOX-1w group (Figure 5E).

The myocardial necrosis markers LDH, CK, CK-MB, and cTnT were positively correlated with *Bacteroides caccae*, *Bifidobacterium pseudolongum*, *Faecalibacterium prausnitzii*, *Mycoplasma hyorhinis*, and *Turicibacter sanguinis*, and negatively correlated with *Roseburia faecis* in DOX-5w group compared with DOX-3w group (Figure 5F).

## 4 Discussion

Our study found that the LVEF, LVFS, and LVPWs significantly decreased, LVIDs significantly increased, and the levels of myocardial necrosis markers increased with the extension of DOX treatment time and the cumulative measurement increased. Our study also found that the abundance of *Firmicutes* significantly decreased whereas that of *Proteobacteria* significantly increased in intestine with the prolonged use of DOX and aggravation of myocardial injury in the DOX-5w group. At the genus level, the abundance of *Clostridium XVIII*, *Butyricimonas*, and *Clostridium sensu stricto* in intestine increased significantly with the aggravation of myocardial injury. Further, the abundance of *Roseburia*, *Asaccharobacter*, *Gemmiger*, and *Peptococcus* significantly decreased. At the species level, no difference in intestinal flora was detected in the DOX-3w group compared with the DOX-1w group. However, the mice developed severe HF, EF further decreased, and intestinal flora disorder was significantly aggravated with DOX dose accumulation (DOX-5w group). Thirteen different bacteria were screened in the DOX-5w group compared with the DOX-1w group, among which, the abundance of *Clostridium disporicum, Turicibacter sanguinis, Escherichia, Butyricimonas virosa, Romboutsia sedimentorum, Bacteroides caccae, Staphylococcus cohnii, Mycoplasma hyorhinis, Bifidobacterium pseudolongum*, and *Bacteroides nordii* increased significantly and that of *Roseburia faecis*, *Mucispirillum schaedleri, and Butyricicoccus pullicaecorum* decreased significantly. Six different bacteria were screened in the DOX-5w group compared with the DOX-3w group, among which, the abundance of *Turicibacter sanguinis, Bacteroides caccae, Bifidobacterium pseudolongum, Faecalibacterium prausnitzii*, and *Mycoplasma hyorhinis* significantly increased and that of *Roseburia faecis* significantly decreased. These results showed that the myocardial injury was aggravated, and the composition and abundance of the intestinal flora in mice significantly changed with the DOX dose accumulation.

In addition, the serum metabolites of mice significantly changed in different degrees of DIHF. Bile acids play an essential role in regulating energy homeostasis and lipid and glucose metabolism. Studies have found that secondary bile acid levels are closely related to HF, atrial fibrillation, hypercholesterolemia, coronary heart disease, and various metabolic diseases (Mayerhofer et al., 2017). *In vitro*, the secondary bile acid DCA significantly induced the release of tumor necrosis factor (TNF-*α*) mRNA from macrophages in a dose-dependent manner (Yan et al., 2021). TUDCA is a conjugated bile acid derivative and has anti-apoptosis and neuroprotective activities (Grant and DeMorrow, 2020). Díaz et al. (2021) found that endoplasmic reticulum (ER) stress inhibitor TUDCA could improve cardiac autonomic balance and reduce the incidence of arrhythmias and abnormal respiratory patterns; that is, it improved cardiac function by inhibiting endoplasmic reticulum stress. TUDCA also alleviated paraquat-induced cardiomyocyte dysfunction (Ge et al., 2010). Our research showed that the levels of DCA and TUDCA showed significantly increased in the DOX-5w group compared with the control group, also, they had a significant predictive value in the DOX-5w group. Moreover, the levels of TUDCA increased more significantly in the DOX-3w group (the early stage of myocardial injury) compared with the DOX-1w group. Further research showed that the DCA and TUDCA were positively correlated with *Bifidobacterium pseudolongum, Clostridium cocleatum, Escherichia, Mycoplasma hyorhinis, Romboutsia sedimentorum*, and *Turicibacter sanguinis*, and negatively correlated with *Asaccharobacter celatus, Roseburia faecis*, and *Faecalicoccus pleomorphus* in the DOX-5w group compared with the control group. These results suggested that DOX regulated DCA and TUDCA levels by increasing or decreasing the abundance of specific gut bacteria, thus affecting the occurrence and development of HF.

TMAO is a metabolite of intestinal flora. Previous studies have confirmed that TMAO can accumulate in the heart, kidney, or other tissues, and participate in a series of biochemical reactions, including activating platelet aggregation, increasing the formation of foam cells, inducing inflammatory reactions, and so forth. These reactions can accelerate atherosclerosis (Din et al., 2019; Yousuf et al., 2022), cardiac remodeling, and the progression of chronic kidney disease to some extent. Previous studies showed that the intestinal flora related to TMAO metabolism mainly included *Anaerococcus*, *Clostridium*, *Desulfitobacter*, *Enterococcus*, *Streptococcus*, and *Proteus* (Al-Obaide et al., 2017). Our study also found a significant increase in the levels of TMAO in the DOX-5w mice compared with the control group, also, TMAO had a significant predictive value in the DOX-5w group. In addition, we found that TMAO was positively correlated with *Bifidobacterium pseudolongum, Clostridium cocleatum, Escherichia, Mycoplasma hyorhinis, Romboutsia sedimentorum*, and *Turicibacter sanguinis*, and negatively correlated with *Asaccharobacter celatus, Roseburia faecis, and Faecalicoccus pleomorphus* in the DOX-5w group compared with the control group. These results suggested that DOX regulated TMAO levels by increasing the abundance of trimethylamine-producing bacteria, thus affecting the occurrence and development of HF.

The changes in cardiac energy metabolism can aggravate the development of HF, and the decrease in mitochondrial oxidation capacity is the main reason for the energy deficiency of the heart in the HF state. The decrease in glucose and amino acid oxidation and the increase in ketone oxidation and other factors can cause abnormal changes in mitochondrial energy (Lopaschuk et al., 2021). Amino acids are not only the building blocks of proteins but also intermediate metabolites that drive multiple biosynthetic pathways. In our studies, the levels of cystine and l-histidine increased significantly in DOX-5w group compared with the control group, the levels of l-tryptophan increased significantly in the DOX-3w group and DOX-5w group compared with the Dox-1w group, and indole-3-acrylic acid (a tryptophan metabolite secreted by gut microbiota) increased significantly in the DOX-5w group compared with the control, DOX-1w and DOX-3w groups, suggesting that the amino acid synthesis and utilization disorders aggravate with the DOX accumulation. Further our research showed that the intestinal differential bacteria *Bifidobacterium pseudolongum* and *Clostridium cocleatum* were significantly correlated with l-histidine, and *Bifidobacterium pseudolongum*, *Escherichia*, *Mycoplasma hyorhinis* and *Roseburia faecis* were significantly correlated with cystine in DOX-5w group compared with the control group. Moreover, our research showed that the *Bifidobacterium pseudolongum, Mycoplasma hyorhinis, Turicibacter sanguinis*, *Bacteroides caccae*, and *Roseburia faecis* were significantly correlated with Indole-3-acrylic acid in DOX-5w group compared with the DOX-1w group and DOX-3w group, suggesting that the different gut microbiota affected different amino acid metabolism and subsequently myocardial energy metabolism in different degrees of DIHF.

Our studies also showed that different metabolic pathways with significant enrichment of differential metabolites participated in different degrees of DIHF. The KEGG metabolic pathways, such as bile secretion; tyrosine metabolism; neuroactive ligand-receptor interaction; phenylalanine, tyrosine and tryptophan biosynthesis; aldosterone synthesis and secretion; and tryptophan metabolism were significantly enriched by differential metabolites in DOX-3w and DOX-5w groups compared with the DOX-1w group. In addition, the aldosterone-regulated sodium reabsorption; nicotinate and nicotinamide metabolism; steroid hormone biosynthesis; and primary bile acid biosynthesis also were significantly enriched by differential metabolites in DOX-5w group compared with the DOX-1w group, nicotinate and nicotinamide metabolism; histidine metabolism; neuroactive ligand-receptor interaction; and tryptophan metabolism were significantly enriched by differential metabolites in DOX-5w group compared with the DOX-3w. These results showed that different accumulated doses of DOX induced varying degrees of myocardial injury through different enrichment pathways.

## 5 Conclusion

In conclusion, the intestinal flora and serum metabolic profile changed significantly with the increase in the cumulative dose of DOX. The intestinal flora may participate in different pathophysiological process in DIHF by causing abnormal metabolism and their metabolites downstream signaling pathways. These gut microbiota and serum metabolites can be used as markers of myocardial damage in different degree of DIHF.

## Data Availability

The data presented in the study are deposited in the NCBI Sequence Read Archive repository, accession number: PRJNA1150914.
